# Association of Bariatric Surgery With Vascular Outcomes

**DOI:** 10.1001/jamanetworkopen.2021.15267

**Published:** 2021-07-12

**Authors:** Noyan Gokce, Shakun Karki, Alyssa Dobyns, Elaina Zizza, Emily Sroczynski, Joseph N. Palmisano, Celestina Mazzotta, Naomi M. Hamburg, Luise I. Pernar, Brian Carmine, Cullen O. Carter, Michael LaValley, Donald T. Hess, Caroline M. Apovian, Melissa G. Farb

**Affiliations:** 1 Department of Medicine and Whitaker Cardiovascular Institute, Boston University School of Medicine, Boston, Massachusetts; 2 Department of Public Health, Boston University School of Medicine, Boston, Massachusetts; 3 Department of General Surgery, Boston University School of Medicine, Boston, Massachusetts; 4 Section of Endocrinology, Diabetes, Nutrition, and Weight Management, Department of Medicine, Boston Medical Center and Boston University School of Medicine, Boston, Massachusetts

## Abstract

**Importance:**

Bariatric surgical weight loss is associated with reduced cardiovascular mortality; however, the mechanisms underlying this association are incompletely understood.

**Objectives:**

To identify variables associated with vascular remodeling after bariatric surgery and to examine how sex, race, and metabolic status are associated with microvascular and macrovascular outcomes.

**Design, Setting, and Participants:**

This population-based longitudinal cohort included 307 individuals who underwent bariatric surgery. Participants were enrolled in the bariatric weight loss program at Boston Medical Center, a large, multi-ethnic urban hospital, with presurgical and postsurgical assessments. Data were collected from December 11, 2001 to August 27, 2019. Data were analyzed in September 2019.

**Exposure:**

Bariatric surgery.

**Main Outcomes and Measures:**

Flow-mediated dilation (FMD) and reactive hyperemia (RH) (as measures of macrovascular and microvascular function, respectively) and clinical variables were measured preoperatively at baseline and at least once postoperatively within 12 months of the bariatric intervention.

**Results:**

A total of 307 participants with obesity (mean [SD] age, 42 [12] years; 246 [80%] women; 199 [65%] White; mean [SD] body mass index, 46 [8]) were enrolled in this study. Bariatric surgery was associated with significant weight loss and improved macrovascular and microvascular function across subgroups of sex, race, and traditional metabolic syndrome (mean [SD] pre- vs postsurgery weight: 126 [25] kg vs 104 [25] kg; *P* < .001; mean [SD] pre- vs postsurgery FMD: 9.1% [5.3] vs 10.2% [5.1]; *P* < .001; mean [SD] pre- vs postsurgery RH: 764% [400] vs 923% [412]; *P* < .001). Factors associated with change in vascular phenotype correlated most strongly with adiposity markers and several metabolic variables depending on vascular territory (eg, association of weight change with change in RH: estimate, −3.2; 95% CI, −4.7 to −1.8; association of hemoglobin A_1c_ with change in FMD: estimate, −0.5; 95% CI, −0.95 to −0.05). While changes in macrovascular function among individuals with metabolically healthy obesity were not observed, the addition of biomarker assessment using high-sensitivity C-reactive protein plasma levels greater than 2 mg/dL identified participants with seemingly metabolically healthy obesity who had low-grade inflammation and achieved microvascular benefit from weight loss surgery.

**Conclusions and Relevance:**

The findings of this study suggest that bariatric intervention is associated with weight loss and favorable remodeling of the vasculature among a wide range of individuals with cardiovascular risk. Moreover, differences in arterial responses to weight loss surgery by metabolic status were identified, underscoring heterogeneity in physiological responses to adiposity change and potential activation of distinct pathological pathways in clinical subgroups. As such, individuals with metabolically healthy obesity represent a mixed population that may benefit from more refined phenotypic classification.

## Key Points

**Question:**

Is bariatric surgical weight loss intervention for obesity associated with microvascular and macrovascular outcomes, and are they differentially associated with sex, race, and metabolic status?

**Findings:**

In 307 individuals with obesity who were followed up after bariatric surgery, the procedure was associated with significant weight loss and improvement in both macrovascular and microvascular function across subgroups of sex, race, and traditional metabolic syndrome. Biomarker assessment using high-sensitivity C-reactive protein plasma levels of greater than 2 mg/dL identified individuals with seemingly metabolically healthy obesity and low-grade inflammation who also derived microvascular benefit from weight loss surgery.

**Meaning:**

These findings suggest that the beneficial effects of weight loss surgery extend broadly across sex, race, and certain metabolically healthy subgroups.

## Teaser

This cohort study identifies variables associated with vascular remodeling after bariatric surgery and examines how sex, race, and metabolic status are associated with microvascular and macrovascular outcomes.

## Introduction

The obesity epidemic has become a major health care crisis, and its prevalence continues to surge worldwide in children and adults.^1,2^ Obesity-related deaths are largely driven by cardiovascular diseases,^3,4,5^ and bariatric surgery has emerged as the most effective and sustainable weight loss strategy as well as the sole weight loss intervention shown to reduce cardiovascular mortality.^6,7,8,9,10,11^ Data from our group and others have demonstrated that reduction in cardiovascular risk may occur as a function of reversing vascular endothelial dysfunction and insulin resistance.^12,13,14,15,16^ The objective of our present longitudinal study was to identify variables associated with vascular improvement after bariatric surgery and examine how sex, race, and metabolic status are associated with microvascular and macrovascular outcomes.

## Methods

### Participants and Study Design

We recruited men and women with obesity (body mass index [BMI; calculated as weight in kilograms divided by height in meters squared] ≥35 ) enrolled in the bariatric surgery program at Boston Medical Center from December 11, 2001, to August 27, 2019. Bariatric operations consisted of Roux-en-Y gastric bypass (RYGB) surgery, sleeve gastrectomy (SG), or laparoscopic adjustable gastric band (LAGB) surgery. Exclusion criteria for the study included patients with recent coronary syndromes, congestive heart failure, malignant neoplasm, systemic infection, acute illness, or pregnancy. The Boston University School of Medicine institutional review board approved study protocol, and all participants provided informed consent. This cohort study adhered to the Strengthening the Reporting of Observational Studies in Epidemiology (STROBE) reporting guideline. Study participants were evaluated at a baseline visit before their surgical intervention and at least 1 follow-up evaluation no later than 12 months after their surgical intervention. During their baseline visit, participants were asked to self-identify sex and race, with race based on National Institutes of Health categories of Black or African American, American Indian or Alaska Native, Asian, Native Hawaiian or other Pacific Islander, and White. Participants who did not select any of these 5 categories checked other race. Categories were truncated into White and Black or other race for the analyses. Race data were collected because research has shown that individuals from minority racial/ethnic groups in the United States are disproportionately affected by obesity and its associated comorbidities compared with White individuals. One participant did not designate themselves as male or female and was not included in the sex-stratified analyses. Clinical characteristics, such as antihypertensive and lipid lowering medication use, blood pressure, height, weight, BMI, hip circumference (HC), and waist circumference (WC) were recorded during each study visit, and all biochemical analyses were quantified from fasting blood samples.

We stratified our cohort with obesity by traditional metabolic syndrome as previously described.^17^ Briefly, a metabolically unhealthy obese (MUHO) phenotype was defined by the presence of any 3 of the following traits: abdominal obesity, defined as a waist circumference in men of 102 cm or greater and in women of 88 cm or greater; triglyceride level of 150 mg/dL or greater (to convert to millimoles per liter, multiply by 0.0113); high-density lipoprotein (HDL) cholesterol level of less than 40 mg/dL in men and less than 50 mg/dL in women (to convert to millimoles per liter, multiply by 0.0259); systolic blood pressure of 130 mm Hg or greater, diastolic blood pressure of 85 mm Hg or greater, or use of any hypertension medication; and fasting plasma glucose level of 100 mg/dL or greater if fasting for at least 6 hours (to convert to millimoles per liter, multiply by 0.0555) or drug treatment for elevated blood glucose. Otherwise, participants were classified as having metabolically healthy obesity (MHO). Seven participants were missing 1 of the classifying data points and were removed from this analysis.

### Vascular Function Studies

During each study visit, participants underwent an ultrasound of the forearm brachial artery while in a supine position during a fasting state in a quiet and temperature-controlled room under resting conditions by trained sonographers. Using a Powervision 6000 system (Toshiba Medical), brachial vasomotor responses were examined, as previously described.^12^ Flow-mediated dilation (FMD) is expressed as percentage change of the brachial artery and serves as a measure of endothelium-dependent dilation of the macrovasculature. Briefly, the brachial artery diameter (in millimeters) is recorded at rest and then again 1 minute after a 5-minute cuff occlusion in an upper arm position above the antecubital crease. Pulsed doppler flow velocity (in centimeters per second) at baseline and after cuff deflation served as the quantified measure of reactive hyperemia (RH). Defined as the percentage change in forearm blood flow, RH served as the measure of endothelium-dependent microvascular function. Specific soft tissue landmarks for each digitized frame were identified to ensure that the same arterial segment was imaged at baseline and during the follow-up visit(s). An investigator masked to clinical information performed all offline analyses of digitized end-diastolic images. Intra-observer and inter-observer correlation coefficients for diameter determination are 0.99 and 0.99, respectively, and 0.93 and 0.89 for FMD.^18^

### Statistical Analysis

Analysis for this cohort study was performed in September 2019. Descriptive statistics of clinical characteristics at baseline and postsurgical assessment were examined; all tests were 2-tailed. Presurgical to postsurgical differences were assessed via paired *t* tests and McNemar test for continuous and categorical variables, respectively. Differences in baseline clinical characteristics by sex, race, and metabolic status were assessed via *t* tests and χ^2^ tests, respectively. To incorporate all information from participants with more than 1 follow-up assessment, we used generalized estimating equations (GEEs) with an autoregressive correlation structure to account for within-participant correlation between repeated measures. In an examination of the association between clinical characteristics and vascular measures, GEE was used, modeling change in the clinical characteristics associated with change in vascular measures. Analyses were stratified by sex, race, and metabolic status. All statistical analyses were performed using SAS version 9.4 (SAS Institute).

## Results

A total of 307 participants with obesity (mean [SD] age, 42 [12] years; 246 [80%] women; 199 [65%] White; mean [SD] BMI, 46 [8]) were enrolled into this study. All participants underwent bariatric surgery for weight loss, with 259 (84%) having RYGB and remaining 48 (16%) undergoing an SG or LAGB surgery. Table 1 displays the clinical parameters measured at the pre- and postsurgical visits. Participants had a mean (SD) follow up period of 5.9 (4.1) months and lost 17.5% of their initial body weight at their postsurgical assessment (mean [SD] pre- vs postsurgery weight: 126 [25] kg vs 104 [25] kg; *P* < .001). As expected, improvements in metabolic parameters, such as glucose, insulin, and lipid levels, accompanied weight loss as well as a decrease in the need for medication use during the length of the study.

**Table 1.** Study Population Characteristics

Variable	Mean (SD)		*P* value
	Baseline visit (n = 307)	Postsurgical visit (n = 307)	
BMI	46 (8)	38 (8)	<.001
Waist circumference, cm	125 (18)	109 (19)	<.001
Weight, kg	126 (25)	104 (25)	<.001
Glucose, mg/dL	111 (51)	94 (25)	<.001
Insulin, μIU/ml	16 (8)	8 (5)	<.001
HOMA-IR	5.4 (6.7)	2.2 (2.9)	<.001
Triglycerides, mg/dL	121 (70)	99 (38)	<.001
Total cholesterol, mg/dL	184 (41)	167 (35)	<.001
HDL cholesterol, mg/dL	47 (11)	43 (13)	<.001
LDL cholesterol, mg/dL	113 (33)	104 (30)	<.001
HbA_1c_, %	6.2 (1.6)	5.5 (0.9)	<.001
hs-CRP, mg/dL	9.9 (9.9)	5.8 (7.8)	<.001
Antihypertensive medication use, No. (%)	161 (53)	76 (25)	<.001
Lipid-lowering medication use, No. (%)	62 (20)	20 (7)	<.001
Flow-mediated dilation, %	9.1 (5.3)	10.2 (5.1)	<.001
Reactive hyperemia, % change	764 (400)	923 (412)	<.001

Abbreviations: BMI, body mass index (calculated as weight in kilograms divided by height in meters squared); HbA_1c_, hemoglobin A_1c_\; HDL, high-density lipoprotein; HOMA-IR, Homeostatic Model Assessment for Insulin Resistance; hs-CRP, high-sensitivity C-reactive protein; LDL, low-density lipoprotein.

SI conversion factors: To convert insulin to picomoles per liter, multiply by 6.945; glucose to millimoles per liter, multiply by 0.0555; HbA_1c_ to proportion of total hemoglobin, multiply by 0.01; HDL, LDL, and total cholesterol to millimoles per liter, multiply by 0.0259; hs-CRP to milligrams per liter, multiply by 10; and triglycerides to millimoles per liter, multiply by 0.0113.

**Figure 1.**
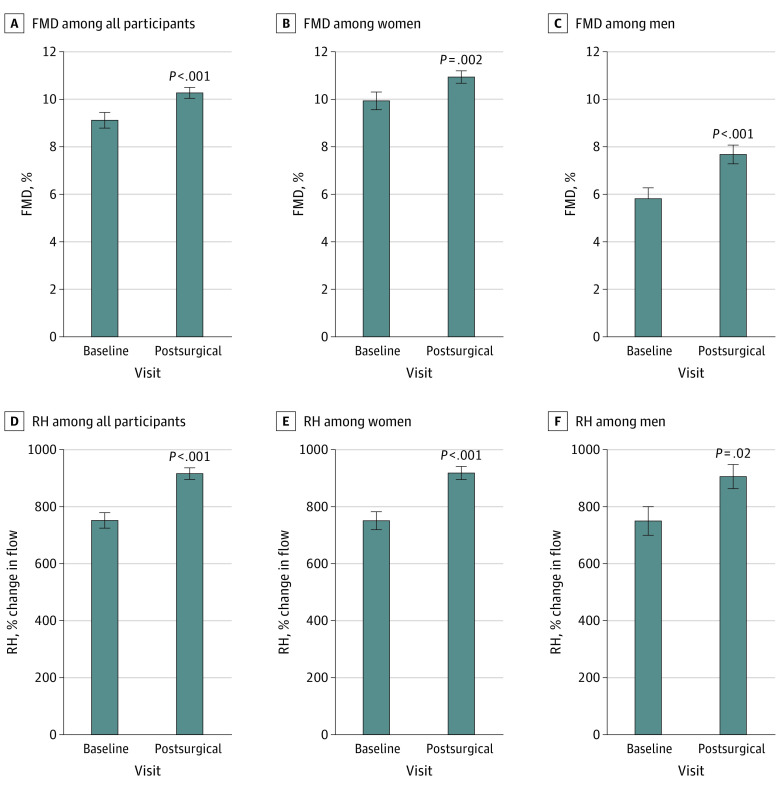
Association of Bariatric Surgery With Vascular Function Data are presented as mean (SEM). FMD indicates flow-mediated dilation; RH, reactive hyperemia.

Both endothelium-dependent FMD and RH were improved after surgery, suggesting functional benefits to both the macrovasculature and microvasculature (mean [SD] pre- vs postsurgery FMD: 9.1% [5.3] vs 10.2% [5.1]; *P* = .001; mean [SD] RH change: 764% [400] vs 923% [412]; *P* < .001) (Figure 1A and Figure 1D). Clinical factors associated with improved RH were the change in weight, BMI, WC, HC and HDL (eg, association of weight change with change in RH: estimate, −3.2; 95% CI, −4.7 to −1.8), while improvement in hemoglobin A_1c_ (HbA_1c_) was associated with change in FMD (estimate, −0.5; 95% CI, −0.95 to −0.05) (Table 2). In contrast, surgical procedure type was not associated with vascular end points. Of note, vascular function improved despite discontinuation of medications known to favorably affect endothelial function; thus, the overall cumulative effect of weight loss on vascular function may have been moderated by stoppage of vasculoprotective agents owing to clinical reasons, such as normotension.

**Table 2.** Change in Clinical and Anthropometric Measures Associated With the Change in Vascular Measures

Variable	GEE estimate (95% CI)^a^					
	Entire cohort (n = 307)		Women (n = 246)		Men (n = 60)	
	Change in FMD	Change in RH	Change in FMD	Change in RH	Change in FMD	Change in RH
Change in weight	−0.01 (−0.028 to 0.009)	−3.2 (−4.7 to −1.8)^b^	−0.02 (−0.04 to 0.006)	−3.6 (−5.4 to −1.8)^b^	0.005 (−0.01 to 0.02)	−2.4 (−4.9 to 0.09)
Change in body mass index	−0.05 (−0.16 to 0.07)	−19.6 (−28.7 to −10.6)^b^	−0.09 (−0.23 to 0.06)	−20.7 (−31.4 to −10.0)^b^	0.03 (−0.10 to 0.16)	−15.4 (−32.9 to 2.2)
Change in waist circumference	−0.06 (−0.19 to 0.06)	−20.4 (−30.0 to −10.7)^b^	−0.07 (−0.2 to 0.08)	−19.7 (−31.2 to −8.2)^b^	−0.07 (−0.21 to 0.08)	−20.8 (−37.9 to −3.7)^c^
Change in hip circumference	−0.025 (−0.15 to 0.09)	−28.8 (−39.5 to −18.0)^b^	−0.08 (−0.2 to 0.05)	−27.5 (−40.3 to −14.6)^b^	−0.03 (−0.20 to 0.13)	−32.7 (−47.4 to −18.1)^b^
Change in total cholesterol	−0.003 (−0.018 to 0.01)	0.35 (−0.9 to 1.7)	−0.005 (−0.02 to 0.01)	0.60 (−0.81 to 2.0)	0.007 (−0.02 to 0.03)	−0.73 (−3.9 to 2.5)
Change in HDL cholesterol	0.005 (−0.04 to 0.05)	5.4 (1.3 to 9.6)^c^	0.006 (−0.04 to 0.06)	5.8 (1.4 to 10.3)^c^	0.08 (−0.02 to 0.17)	−0.75 (−17.0 to 15.5)
Change in LDL cholesterol	−0.002 (−0.02 to 0.02)	−0.9 (−2.5 to 0.6)	−0.006 (−0.03 to 0.02)	−0.74 (−2.4 to 0.9)	0.008 (−0.02 to 0.03)	−1.7 (−5.4 to 2.0)
Change in triglycerides	−0.002 (−0.01 to 0.008)	−0.3 (−1.3 to 0.7)	−0.001 (−0.01 to 0.01)	−0.38 (−1.6 to 0.84)	−0.009 (−0.02 to 0.005)	0.05 (−1.7 to 1.6)
Change in glucose	−0.01 (−0.02 to 0.003)	−0.5 (−1.6 to 0.7)	−0.01 (−0.02 to 0.005)	−0.3 (−1.6 to 1.0)	−0.005 (−0.03 to 0.02)	−1.5 (−3.3 to 0.43)
Change in insulin	−0.02 (−0.08 to 0.04)	−7.5 (−16 to 1.1)	−0.02 (−0.10 to 0.06)	−5.8 (−15.7 to 4.1)	0.007 (−0.08 to 0.09)	−14.4 (−29.9 to 1.2)
Change in HOMA-IR	−0.04 (−0.1 to 0.05)	−4.1 (−12.8 to 4.5)	0.008 (−0.12 to 0.10)	−7.3 (−18.0 to 3.4)	−0.06 (−0.12 to 0.005)	0.08 (−9.4 to 9.6)
Change in HbA_1c_	−0.5 (−0.95 to −0.05)^c^	−19.6 (−90.8 to 51.6)	−0.67 (−1.2 to −0.17)^d^	−26.9 (−104.2 to 50.4)	0.4 (−0.8 to 1.7)	68.0 (−91.5 to 227.5)
Change in hs-CRP	0.003 (−0.05 to 0.05)	−4.4 (−10.1 to 1.3)	0.009 (−0.09 to 0.07)	−8.2 (−15.3 to −1.1)^c^	−0.004 (−0.04 to 0.03)	−1.5 (−10.3 to 7.3)

Abbreviations: FMD, flow-mediated dilation; GEE, generalized estimating equations; HbA_1c_, hemoglobin A_1c_; HDL, high-density lipoprotein; HOMA-IR, Homeostatic Model Assessment for Insulin Resistance; hs-CRP, high-sensitivity C-reactive protein; LDL, low-density lipoprotein; RH, reactive hyperemia.

^a^ Data are presented as GEE estimates of association of the change in a clinical variable and vascular measures for 1-unit change in the variable measure.

^b^ *P* < .001.

^c^ *P* < .05.

^d^ *P* < .01.

### Stratification by Sex

Next, we explored whether vascular outcomes over time differed when stratified by sex (eTable 1 in the Supplement). The mean (SD) follow-up period for men and women was 6.0 (4.0) and 5.9 (4.1) months, respectively. Both men and women had similar BMI at baseline and lost a comparable percentage of weight at their postoperative assessment. Women displayed an improvement in FMD and RH following bariatric surgery (mean [SD] FMD: presurgery, 9.9% [5.4] vs postsurgery, 10.9% [4.9]; *P* = .002) (Figure 1B and Figure 1E). Decreased measures of adiposity assessed by weight, BMI, WC, and HC, lower circulating high-sensitivity C-reactive protein (hs-CRP), and increased HDL cholesterol were all associated with improvement in RH, while change in HbA_1c_ was associated with FMD (Table 2). Similarly, we observed a significant improvement in FMD and RH in men at their postoperative assessment (mean [SD] FMD: presurgery, 5.8% [3.3] vs postsurgery, 7.7% [3.9]; *P* < .001) (Figure 1C and Figure 1F), with increased RH associated with decreases in WC and HC (Table 2). As such, the improvement in FMD and RH in men and women was equivalent.

### Stratification by Self-identified Race

**Figure 2.**
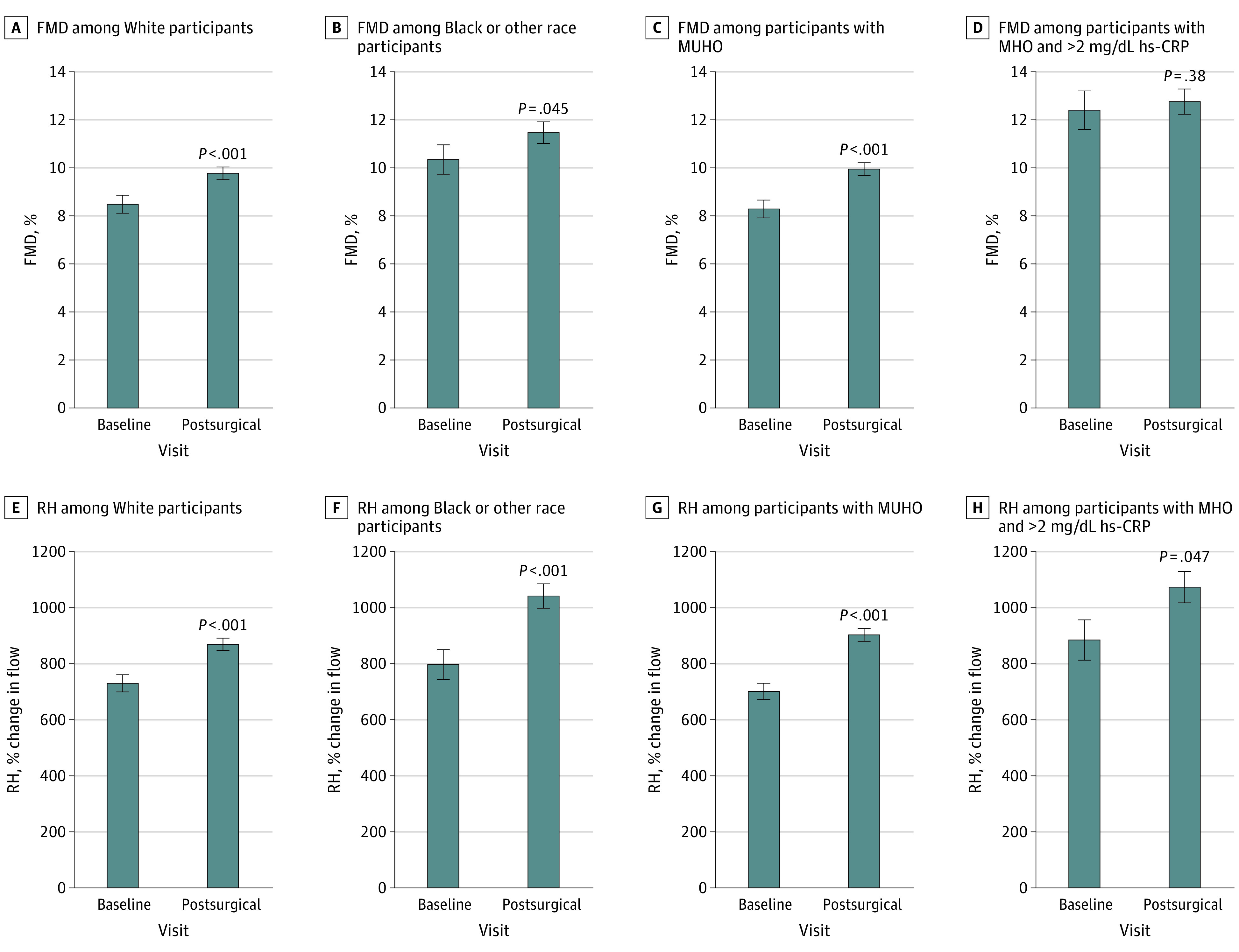
Microvascular and Macrovascular Function Improvement Following Bariatric Surgery by Race and Metabolic Status Data are presented as mean (SEM). FMD indicates flow-mediated dilation; hs-CRP, high sensitivity C-reactive protein (to convert to milligrams per liter, multiply by 10); MHO, metabolically healthy obesity; MUHO, metabolically unhealthy obesity; and RH, reactive hyperemia.

In our cohort, 199 participants (65%) self-identified as White, 75 participants (24%) self-identified as Black, and 33 participants (11%) self-identified as other. Baseline clinical characteristics stratified by race are displayed in eTable 2 in the Supplement. White participants were older and had higher blood levels of triglycerides and cholesterol compared with the rest of the cohort, while BMI was similar across races prior to surgery. As displayed in Figure 2A and Figure 2E, White participants had a significant improvement in FMD (mean [SD] FMD: presurgery, 8.5% [4.9] vs postsurgery, 9.8% [4.7]; *P* < .001) and RH at their postoperative assessment (mean [SD] 6.0 [4.1] months follow-up time). Changes in adiposity measures and hs-CRP were negatively associated with RH, and improvements in circulating HDL was associated positively with microvascular function (Table 3). Black participants and those who identified as other also displayed favorable macrovascular and microvascular outcomes at their postoperative visit (mean [SD] FMD: presurgery, 10.3% [5.7] vs postsurgery, 11.5% [5.2]; *P* = .045; mean [SD] follow-up time, 5.7 [4.0] months) (Figure 2B and Figure 2F), and factors associated with microvascular change were similar across races. In contrast, amelioration in triglycerides, glucose control, and insulin resistance were associated with the improvement in FMD only in participants who identified as belonging to a minority racial group, highlighting that determinants of vascular remodeling following weight loss surgery may differ between races.

**Table 3.** Change in Clinical and Anthropometric Measures Associated With Change in Vascular Measures Stratified by Race and Metabolic Status

Variable	GEE estimate (95% CI)^a^						
	White (n = 199)		Black or other race (n = 108)		MUHO (n = 222)		MHO with hs-CRP level ≥2 mg/dL (n = 53)
	Change in FMD	Change in RH	Change in FMD	Change in RH	Change in FMD	Change in RH	Change in RH
Change in weight	−0.01 (−0.03 to 0.01)	−3.1 (−4.6 to −1.6)^b^	−0.006 (−0.05 to 0.03)	−4.6 (−8.9 to −0.3)^c^	−0.01 (−0.04 to 0.01)	−2.6 (−4.0 to −1.1)^b^	−5.7 (−11 to −0.5)^c^
Change in body mass index	−0.07 (−0.20 to 0.06)	−19.2 (−28.6 to −9.9)^b^	0.01 (−0.26 to 0.28)	−27.7 (−54.7 to −0.7)^c^	−0.08 (−0.2 to 0.06)	−15.1 (−24.0 to −6.2)^b^	−39.3 (−75.3 to −3.3)^c^
Change in waist circumference	−0.07 (−0.19 to 0.06)	−19.9 (−30.1 to −9.6)^b^	−0.06 (−0.3 to 0.2)	−25.3 (−47.3 to −3.3)^c^	−0.09 (−0.2 to 0.05)	−15.8 (−25.7 to −5.8)^d^	−40.9 (−76.3 to −5.7)^c^
Change in hip circumference	0.04 (−0.11 to 0.19)	−27.5 (−39.4 to −15.5)^b^	−0.21 (−0.49 to 0.07)	−36.6 (−62.2 to −11.0)^d^	−0.06 (−0.2 to 0.08)	−22.2 (−32.8 to −11.6)^b^	−58.8 (−87.3 to −30.3)^b^
Change in total cholesterol	−0.0003 (−0.02 to 0.02)	0.76 (−0.56 to 2.1)	−0.01 (−0.05 to 0.03)	−1.1 (−4.5 to 2.3)	−0.005 (−0.02 to 0.01)	−0.09 (−1.4 to 1.2)	3.2 (−1.1 to 7.4)
Change in HDL cholesterol	−0.001 (−0.06 to 0.06)	7.8 (3.6 to 12.1)^b^	0.03 (−0.025 to 0.09)	2.3 (−3.2 to 7.9)	0.008 (−0.05 to 0.06)	3.7 (−0.4 to 7.8)	10.9 (1.8 to 19.9)^c^
Change in LDL cholesterol	−0.002 (−0.02 to 0.016)	−0.87 (−2.4 to 0.69)	−0.002 (−0.05 to 0.05)	−1.1 (−4.9 to 2.7)	−0.005 (−0.02 to 0.01)	−1.2 (−2.7 to 0.3)	1.7 (−3.7 to 7.0)
Change in triglycerides	0.005 (−0.007 to 0.02)	0.23 (−0.82 to 1.3)	−0.01 (−0.03 to −0.002)^c^	−1.5 (−2.6 to −0.26)^c^	−0.002 (−0.01 to 0.01)	−0.05 (−1.1 to 1.0)	1.9 (−2.6 to 6.3)
Change in glucose	0.003 (−0.01 to 0.02)	0.21 (−0.85 to 1.26)	−0.03 (−0.04 to −0.009)^d^	−0.94 (−2.8 to 0.9)	−0.01 (−0.02 to 0.003)	−0.35 (−1.5 to 0.8)	13.9 (6.9 to 20.9)^b^
Change in insulin	0.003 (−0.07 to 0.08)	−6.8 (−14.0 to 0.43)	−0.09 (−0.19 to 0.005)	−14.6 (−41.9 to 12.8)	−0.03 (−0.1 to 0.04)	−5.9 (−14.5 to 2.7)	0.6 (−55.4 to 56.5)
Change in HOMA-IR	0.03 (−0.07 to 0.13)	−5.2 (−16.1 to 5.7)	−0.17 (−0.3 to −0.04)^c^	−1.4 (−15.7 to 12.9)	−0.04 (−0.1 to 0.06)	−1.9 (−10.1 to 6.3)	42.9 (−145.9 to 231.7)
Change in HbA_1c_	0.27 (−0.54 to 1.1)	35.2 (−43.9 to 114.2)	−0.82 (−1.4 to −0.2)^d^	−39.0 (−131.2 to 53.2)	−0.47 (−0.9 to 0.002)	−11.8 (−87.2 to 63.6)	161.8 (−167.9 to 491.5)
Change in hs-CRP	0.02 (−0.07 to 0.03)	−7.4 (−13.5 to −1.2)^c^	0.07 (−0.07 to 0.2)	5.4 (−6.2 to 17.1)	0.001 (−0.05 to 0.05)	−4.6 (−10.5 to 1.2)	31.2 (14.3 to 48.1)^b^

Abbreviations: FMD, flow-mediated dilation; GEE, generalized estimating equations; HbA_1c_, hemoglobin A_1c_; HDL, high-density lipoprotein; HOMA-IR, Homeostatic Model Assessment for Insulin Resistance; hs-CRP, high-sensitivity C-reactive protein; LDL, low-density lipoprotein; MHO, metabolically healthy obesity; MUHO, metabolically unhealthy obesity; RH, reactive hyperemia.

SI conversion factor: To convert hs-CRP to milligrams per liter, multiply by 10.

^a^ Data are presented as GEE estimates of association of the change in a clinical variable and vascular measures for 1-unit change in the variable measure.

^b^ *P* < .001.

^c^ *P* < .05.

^d^ *P* < .01.

### Stratification by Traditional Metabolic Syndrome

When we stratified our cohort by traditional metabolic syndrome criteria, 222 participants (74%) had metabolic syndrome at the baseline visit and were classified as metabolically unhealthy with obesity (MUHO) vs 78 (26%) who were metabolically healthy with obesity (MHO). By definition, participants with MUHO had more cardiovascular risk factors, including greater WC and higher prevalence of diabetes, hypertension, and hyperlipidemia, compared with those with MHO. Additionally, participants with MUHO were older, and the group had fewer women than the group with MHO, but measures of adiposity were comparable at baseline (eTable 3 in the Supplement). Participants with MUHO had a mean (SD) follow-up time of 5.9 (4.1) months, and those with MHO had a mean (SD) follow up time of 5.9 (4.1) months. Following bariatric surgery, participants with MUHO and MHO had similar degrees of weight loss and significant improvements in clinical variables, such as HbA_1c_ levels, Homeostatic Model Assessment for Insulin Resistance score, and lipid and hs-CRP levels, were observed, despite participants with MHO having a more favorable presurgical metabolic profile.

However, vascular outcomes after surgical intervention differed between the MUHO and MHO groups. In the MUHO group, both macrovascular and microvascular function improved (mean [SD] FMD: presurgery, 8.3% [5.1] vs postsurgery, 9.9% [5.0]; *P* < .001) (Figure 2C and Figure 2G) with change in weight, BMI, WC, and HC associated with microvascular function (Table 3). Among participants with MHO, there was no significant alteration in FMD or RH after surgery. Therefore, we excluded all participants with MHO with circulating hs-CRP levels of 2 mg/dL or lower, creating an MHO group that was further classified as exhibiting chronic inflammation (hs-CRP >2 mg/dL) as an additional cardiovascular disease risk factor.^19^ Comparable differences in clinical parameters between participants with MHO and hs-CRP levels greater than 2 mg/dL and participants with MUHO were observed (eTable 4 in the Supplement). In this subset of participants with MHO, RH improved significantly (mean [SD] RH: presurgery, 885% [413] vs postsurgery, 1074% [391]; *P* = .047), while FMD remained unaltered at the postoperative assessment (mean [SD] follow-up time, 5.9 [4.1] months) (Figure 2D and 2H). These findings suggest that even participants with seemingly metabolically healthy obesity, when stratified by elevated inflammatory biomarkers, such as hs-CRP, experienced vascular benefit after bariatric weight loss.

## Discussion

To our knowledge, this study represents the largest longitudinal bariatric surgical cohort that examined both FMD and RH as measures of macrovascular and microvascular function, respectively. We found that both vascular outcomes, which have been clinically validated as independent predictors of cardiovascular risk,^20,21,22^ significantly improved following bariatric intervention and track changes in anthropometric measures and metabolic risk factors, depending on vascular territory. In smaller, previous studies, our group and others have observed improvement in subclinical atherosclerosis and vascular health following weight loss.^12,13,23,24,25^ Our subset analyses identified differences in arterial responses to weight loss surgery by metabolic status, underscoring heterogeneity in physiological response to adiposity change and potential activation of distinct pathological pathways in obesity subgroups. Moreover, by incorporating inflammatory biomarker assessment, we found that participants with seemingly MHO and signs of chronic low-grade inflammation experienced vascular benefit after weight loss, touching on a growing area of interest and controversy in the field that warrants further investigation.

Bariatric surgery has been shown to improve metabolism, reduce morbidity and mortality, and favorably remodel the vasculature.^8,13,14,15,16,26,27,28,29,30,31,32^ Clinical studies have observed improvement in FMD and carotid intima thickening (CIMT) after bariatric operations,^13,16,26,31,33,34,35^ and amelioration of metabolic syndrome, type 2 diabetes, and insulin resistance have been associated with enhanced microvascular function.^14,30,36,37,38,39^ Thus, our findings extend these previous reports that involved smaller cohorts and shorter follow-up periods.^13,26,33,35^ In our study, while we identified several traditional risk factors as associated with FMD and RH, these associations were modest or tracked weakly, particularly for macrovascular outcomes, consistent with some prior reports.^13,16,33^ In this regard, recent mortality data from the Swedish Obesity Study (SOS) showed that while most deaths were cardiovascular, treatment benefits and increased survival following bariatric surgery were actually even across subgroups of traditional risk factors, such as hypertension and cholesterol levels.^40^ These data, in conjunction with our present findings, highlight significant knowledge gaps in identifying mechanistic regulators of vascular physiology with weight change and underscore our need to search beyond the usual suspects, potentially considering the role of adipocytokines and genetic variability. Although bariatric surgery type may influence the extent of metabolic recovery,^41,42,43,44^ we did not identify a specific surgical approach that was differentially associated with vascular function.

While bariatric surgery is an effective strategy to combat obesity, there is considerable variability in comorbidity remission across individuals.^45,46^ This has generated clinical discussions regarding whether bariatric surgery can serve as a one size fits all approach in the treatment of obesity or whether tailored strategies are needed to improve outcomes in specific patient populations. For decades, clinical studies have consistently found that women are more likely to pursue bariatric surgery than men.^47,48,49,50^ Moreover, although men may have greater positive postoperative psychological outcomes,^47^ male sex is independently associated with major postoperative complications, mortality, and lower weight loss.^47,50,51,52^ Very limited data exist surrounding the association of bariatric surgery with vascular function with respect to sex, with small studies showing changes in CIMT after surgery.^53,54^ Our finding that both sexes had comparable percentage of weight loss and derived similar improvement in both macrovascular and microvascular function following weight loss surgery is of public health importance. Moreover, some inconsistent observations within the literature surrounding the differential outcomes of bariatric surgery among men and women underscores the need for sex-balanced studies.

Minority populations in the United States bear a disproportionate burden of the obesity epidemic and its associated comorbidities and tend to undergo fewer bariatric procedures compared with non-Hispanic White populations, which may be associated with socioeconomic factors, limited access to health care, and/or insurance options.^55,56^ In addition, recent studies have identified race as an important factor associated with weight fluctuations, remission of obesity-related comorbidities, lower weight loss, and higher rates of hospital resource utilization and mortality after bariatric surgery.^57,58,59,60,61,62,63^ In our current study, reversal of microvascular and macrovascular dysfunction was evident in both White participants and participants who identified as a minority racial/ethnic group, but the factors associated with vascular remodeling differed between races. Minority populations have been associated with lower hypertension remission and higher mortality rates,^56,58,59,62,63^ which may partly relate to abnormalities in vascular tone. Racial differences may exist in their anatomical and physiological properties, which warrant further investigation.

Finally, the notion of whether patients with MHO should be approached differently remains a controversial clinical debate.^64^ Recent data suggest that these individuals may be in a transient physiological state along a progression to obesity-related diseases and should seek therapeutic lifestyle changes.^65,66^ Participants with obesity frequently display histological evidence of chronic low-grade inflammation in various organ systems, particularly hepatic and adipose tissues as well as plasma elevation in hs-CRP^19^ that may contribute to cardiometabolic diseases. In the present study, we used a more stringent definition of MHO, stratifying subjects by hs-CRP level as an inflammatory biomarker that is independently associated with vascular events and improves global classification in cardiovascular disease.^19,67^ In participants with MHO and elevated hs-CRP levels, we observed significant improvement in microvascular function following bariatric intervention, which was also associated with lower hs-CRP levels. These support the findings from the SOS study, suggesting that bariatric surgery is associated with reduced risk of microvascular complications across subsets of participants with obesity and varying degrees of metabolic disease.^36^ Thus, the notion of incorporating additional clinical markers to identify participants with MHO at higher risk may have clinical value.

### Limitations

This study has limitations. First, most study participants were women, which reflects the general clinical practice and known sex differences in populations that seek bariatric treatments.^37,49,50,68^ Second, the study was observational and not randomized, and most surgeries were RYGB operations that were determined clinically. Third, racial categories were based on self-identification rather than ancestry informative markers. However, these are counterbalanced by the relatively large sample for this type of prospective clinical investigation involving validated vascular physiological outcomes. Fourth, we acknowledge that across the analyses conducted here, there were many hypothesis tests performed. We have not corrected the significance level to account for the multiple testing performed in the study; thus, there is the potential for some false-positive findings. Ideally, notwithstanding the challenges, these results should be validated in further studies.

## Conclusions

In conclusion, the findings of this study suggest that bariatric surgery is associated with reductions in weight, amelioration of cardiovascular risk factors, and improvement in vascular endothelial phenotype. Improvements in microvascular function, in particular, were observed across all subgroups of sex, race, and metabolic syndrome, while variability in macrovascular changes were observed within subcategories of our cohort. Lastly, factors associated with vascular change were largely modest using known traditional clinical variables, exposing our limited knowledge of mechanisms that govern vascular physiology in obesity and underscoring the crucial need for further investigative studies in this field.

## Notes

Supplement. **eTable 1. **Clinical Characteristics Stratified by Sex **eTable 2. **Clinical Characteristics Stratified by Race **eTable 3. **Clinical Characteristics of Participants With Metabolically Unhealthy Obesity (MUHO) vs Metabolically Healthy Obesity (MHO) **eTable 4. **Clinical Characteristics of Participants With Metabolically Unhealthy Obesity (MUHO) vs Metabolically Healthy Obesity (MHO) and High-Sensitivity C-Reactive Protein (hs-CRP) Levels Greater Than 2 mg/dL
