# Supplementary material for: Association of Bariatric Surgery With Vascular Outcomes
Source: JAMA Netw Open. 2021 Jul 12;4(7):e2115267. doi: 10.1001/jamanetworkopen.2021.15267 (PMC8276087; doi:10.1001/jamanetworkopen.2021.15267)
Supplement: Supplement. — eTable 1. Clinical Characteristics Stratified by Sex eTable 2. Clinical Characteristics Stratified by Race eTable 3. Clinical Characteristics of Participants With Metabolically Unhealthy Obesity (MUHO) vs Metabolically Healthy Obesity (MHO) eTable 4. Clinical Characteristics of Participants With Metabolically Unhealthy Obesity (MUHO) vs Metabolically Healthy Obesity (MHO) and High-Sensitivity C-Reactive Protein (hs-CRP) Levels Greater Than 2 mg/dL [file jamanetwopen-e2115267-s001.pdf]

Supplemental Online Content

Gokce N, Karki S, Dobyns A, et al. Association of bariatric surgery with vascular outcomes. *JAMA Netw Open.* 2021;4(7):e2115267. doi:10.1001/jamanetworkopen.2021.15267

eTable 1. Clinical Characteristics Stratified by Sex

eTable 2. Clinical Characteristics Stratified by Race

eTable 3. Clinical Characteristics of Participants With Metabolically Unhealthy Obesity (MUHO) vs Metabolically Healthy Obesity (MHO)

eTable 4. Clinical Characteristics of Participants With Metabolically Unhealthy Obesity (MUHO) vs Metabolically Healthy Obesity (MHO) and High-Sensitivity C-Reactive Protein (hs-CRP) Levels Greater Than 2 mg/dL

This supplemental material has been provided by the authors to give readers additional information about their work.

© 2021 Gokce N et al. *JAMA Network Open.*

eTable 1. Clinical Characteristics Stratified by Sex

Clinical Parameter	Men (n=60)	Women (n=246)	P Value
**Age, y**	48 (11)	41 (12)	<0.001
**Body Mass Index, kg/m^2**	46 (8)	46 (8)	0.786
**Waist circumference, cm**	135 (16)	123 (17)	<0.001
**Weight, kg**	144 (25)	122 (24)	<0.001
**Glucose, mg/dl**	114 (47)	111 (52)	0.649
**Insulin, µIU/ml**	17 (8)	15 (8)	0.154
**HOMA-IR**	6 (8)	5 (6)	0.325
**Triglycerides, mg/dl**	130 (81)	119 (66)	0.341
**Total Cholesterol, mg/dl**	179 (43)	185 (40)	0.380
**HDL-C, mg/dl**	41 (8)	49 (13)	<0.001
**LDL-C, mg/dl**	112 (34)	112 (33)	0.922
**HbA1C, %**	6.2 (1.4)	6.2 (1.7)	0.785
**hs-C Reactive Protein, mg/dl**	9.2 (13.3)	9.9 (8.7)	0.725
**Flow mediated dilation, %**	5.8 (3.3)	10.1 (5.3)	<0.001
**Reactive Hyperemia, % change**	761 (344)	752 (413)	0.891

Descriptive statistics that are normally distributed are presented as mean with standard deviation (SD), and non-normally distributed data are presented as medians with interquartile ranges (IQRs). Abbreviations: HOMA-IR: Homeostatic Model Assessment of Insulin Resistance; HDL-C: High-density lipoprotein cholesterol; LDL-C: Low-density lipoprotein cholesterol; HbA1C: Hemoglobin A1C.

© 2021 Gokce N et al. *JAMA Network Open.*

eTable 2. Clinical Characteristics Stratified by Race

Clinical Parameter	White (n=199)	Black or other race (n=108)	P value
**Female, %**	77	86	0.063
**Age, y**	44 (12)	40 (11)	0.006
**Body Mass Index, kg/m^2**	46 (8)	45 (7)	0.356
**Waist circumference, cm**	127 (18)	121 (16)	0.009
**Weight, kg**	127 (27)	124 (23)	0.306
**Glucose, mg/dl**	111 (46)	112 (59)	0.857
**Insulin, µIU/ml**	16 (8)	15 (8)	0.724
**HOMA-IR**	5 (7)	6 (7)	0.857
**Triglycerides, mg/dl**	129 (65)	106 (75)	0.006
**Total Cholesterol, mg/dl**	187 (42)	176 (37)	0.022
**HDL-C, mg/dl**	47 (13)	47 (12)	0.851
**LDL-C, mg/dl**	115 (35)	106 (29)	0.010
**HbA1C, %**	6.1 (1.4)	6.3 (1.9)	0.265
**hs-C Reactive Protein, mg/dl**	9.6 (10.3)	10.5 (9.0)	0.485
**Flow mediated dilation, %**	8.7 (4.9)	10.3 (5.6)	0.014
**Reactive Hyperemia, % change**	739 (378)	789 (440)	0.368

Descriptive statistics that are normally distributed are presented as mean with standard deviation (SD), and non-normally distributed data are presented as medians with interquartile ranges (IQRs). Abbreviations: HOMA-IR: Homeostatic Model Assessment of Insulin Resistance; HDL-C: High-density lipoprotein cholesterol; LDL-C: Low-density lipoprotein cholesterol; HbA1C: Hemoglobin A1C.

© 2021 Gokce N et al. *JAMA Network Open.*

eTable 3. Clinical Characteristics of Participants With Metabolically Unhealthy Obesity (MUHO) vs Metabolically Healthy Obesity (MHO)

Clinical Parameter	MUHO (n=222)	MHO (n=78)	P value
**Female, %**	77	88	0.023
**Age, y**	45 (12)	37 (11)	<0.001
**Body Mass Index, kg/m^2**	46 (8)	45 (6)	0.629
**Waist circumference, cm**	127 (17)	119 (17)	0.001
**Weight, kg**	126 (26)	123 (22)	0.254
**Glucose, mg/dl**	120 (57)	89 (10)	<0.001
**Insulin, µIU/ml**	16 (8)	13 (7)	<0.001
**HOMA**	6.4 (7.6)	2.8 (1.5)	<0.001
**Triglycerides, mg/dl**	131 (76)	91 (33)	<0.001
**Total Cholesterol, mg/dl**	182 (42)	186 (35)	0.488
**HDL-C, mg/dl**	46 (13)	51 (12)	0.003
**LDL-C, mg/dl**	110 (33)	117 (31)	0.134
**HbA1C, %**	6.5 (1.8)	5.3 (0.4)	<0.001
**hs-C Reactive Protein, mg/dl**	10 (10)	10 (9)	0.818
**Flow mediated dilation, %**	8.4 (5.1)	11.9 (4.9)	<0.001
**Reactive Hyperemia, % change**	695 (373)	927 (431)	<0.001

Descriptive statistics that are normally distributed are presented as mean with standard deviation (SD), and non-normally distributed data are presented as medians with interquartile ranges (IQRs). Abbreviations: MUHO: Metabolically unhealthy obese; MHO: Metabolically healthy obese; HOMA-IR: Homeostatic Model Assessment of Insulin Resistance; HDL-C: High-density lipoprotein cholesterol; LDL-C: Low-density lipoprotein cholesterol; HbA1C: Hemoglobin A1C.

© 2021 Gokce N et al. *JAMA Network Open.*

eTable 4. Clinical Characteristics of Participants With Metabolically Unhealthy Obesity (MUHO) vs Metabolically Healthy Obesity (MHO) and High-Sensitivity C-Reactive Protein (hs-CRP) Levels Greater Than 2 mg/dL

Clinical Parameter	MUHO (n=222)	MHO with >2 mg/dl hs-CRP (n=53)	P value
**Female, %**	77	89	0.001
**Age, y**	45 (12)	36 (10)	<0.001
**Body Mass Index, kg/m^2**	46 (8)	45 (6)	0.233
**Waist circumference, cm**	127 (17)	119 (16)	0.002
**Weight, kg**	126 (26)	121 (20)	0.123
**Glucose, mg/dl**	120 (57)	89 (7)	<0.001
**Insulin, μIU/ml**	16 (8)	13 (7)	0.003
**HOMA**	6.4 (7.6)	2.8 (1.5)	<0.001
**Triglycerides, mg/dl**	131 (76)	90 (31)	<0.001
**Total Cholesterol, mg/dl**	182 (42)	184 (34)	0.727
**HDL-C, mg/dl**	46 (13)	48 (12)	0.191
**LDL-C, mg/dl**	110 (33)	118 (31)	0.106
**HbA1C, %**	6.5 (1.8)	5.3 (0.4)	<0.001
**hs-C Reactive Protein, mg/dl**	10 (10)	11 (9)	0.619
**Flow mediated dilation, %**	8.4 (5.1)	12.6 (5.3)	<0.001
**Reactive Hyperemia, % change**	695 (373)	868 (409)	0.011

Descriptive statistics that are normally distributed are presented as mean with standard deviation (SD), and non-normally distributed data are presented as medians with interquartile ranges (IQRs). Abbreviations: MUHO: Metabolically unhealthy obese; MHO: Metabolically healthy obese; hs-CRP: High sensitivity C reactive protein; HOMA-IR: Homeostatic Model Assessment of Insulin Resistance; HDL-C: High-density lipoprotein cholesterol; LDL-C: Low-density lipoprotein cholesterol; HbA1C: Hemoglobin A1C.

© 2021 Gokce N et al. *JAMA Network Open.*
